# Clinical Characteristics, Prognostic Factor and a Novel Dynamic Prediction Model for Overall Survival of Elderly Patients With Chondrosarcoma: A Population-Based Study

**DOI:** 10.3389/fpubh.2022.901680

**Published:** 2022-06-30

**Authors:** Yuexin Tong, Yuekai Cui, Liming Jiang, Yangwei Pi, Yan Gong, Dongxu Zhao

**Affiliations:** ^1^Department of Orthopaedics, China-Japan Union Hospital of Jilin University, Changchun, China; ^2^Wenzhou Medical University, Wenzhou, China

**Keywords:** chondrosarcoma, prognostic factors, elderly, nomogram, SEER database

## Abstract

**Background:**

Chondrosarcoma is the most common primary bone sarcoma among elderly population. This study aims to explore independent prognostic factors and develop prediction model in elderly patients with CHS.

**Methods:**

This study retrospectively analyzed the clinical data of elderly patients diagnosed as CHS between 2004 and 2018 from the Surveillance, Epidemiology, and End Results (SEER) database. We randomly divided enrolled patients into training and validation group, univariate and multivariate Cox regression analyses were used to determine independent prognostic factors. Based on the identified variables, the nomogram was developed and verified to predict the 12-, 24-, and 36-month overall survival (OS) of elderly patients with CHS. A k-fold cross-validation method (*k*=10) was performed to validate the newly proposed model. The discrimination, calibration and clinical utility of the nomogram were assessed using the Harrells concordance index (C-index), receiver operating characteristic (ROC) curve and the area under the curve (AUC), calibration curve, decision curve analysis (DCA), the integrated discrimination improvement (IDI) and net reclassification index (NRI). Furthermore, a web-based survival calculator was developed based on the nomogram.

**Results:**

The study finally included 595 elderly patients with CHS and randomized them into the training group (419 cases) and validation group (176 cases) at a ratio of 7:3. Age, sex, grade, histology, M stage, surgery and tumor size were identified as independent prognostic factors of this population. The novel nomogram displayed excellent predictive performance, which can be accessible by https://nomoresearch.shinyapps.io/elderlywithCHS/, with a C-index of 0.800 for the training group and 0.789 for the validation group. The value AUC values at 12-, 24-, and 36-month of 0.866, 0.855, and 0.860 in the training group and of 0.839, 0.856, and 0.840 in the validation group, respectively. The calibration curves exhibited good concordance from the predicted survival probabilities to actual observation. The ROC curves, IDI, NRI, and DCA showed the nomogram was superior to the existing AJCC staging system.

**Conclusion:**

This study developed a novel web-based nomogram for accurately predicting probabilities of OS in elderly patients with CHS, which will contribute to personalized survival assessment and clinical management for elderly patients with CHS.

## Introduction

Chondrosarcoma (CHS), which is characterized by the formation of a cartilaginous matrix, is the most common malignant bone tumor in geriatric population (1). It accounts for ~30% of all primary bone neoplasms (2, 3). In the majority of cases, due to indolent tumor growth behavior and appropriate treatment (mainly complete surgical excision), patients with CHS have a generally favorable survival outcome, with an overall 5-year survival rate of around 70% (4). Nevertheless, the prognosis of elderly patients remains dismal, a retrospective analysis revealed that the 5-year survival rate of patients with CHS older than 60 years was significantly lower than that of patients younger 60 years (5). In addition, unlike osteosarcoma and Ewing's sarcoma, CHS primarily affects adults older than 50 years and the incidence rate increases steadily with age (6, 7). This confirms that elderly patients are an important subgroup of the overall CHS entity that deserves significant attention.

Survival outcomes for cancer patients are influenced collectively by multiple factors, including clinicopathological factors and treatment strategies. Several previous studies have investigated the prognostic factors for CHS (8–10). In this regard, Song et al. developed a nomogram for predicting survival for patients with CHC (11). Another relevant study conducted by Wang et al. specially determined predictors of the survival among CHC patients with metastatic disease at diagnosis (12). Whereas, almost all of these studies focused on the entire entity of patients with CHS rather than on the specific elderly population. Patients of advanced age are often accompanied comorbidity, organ dysfunction and immunosenescence, which let elderly patients experience more treatment-related toxicity and poor prognosis (13). Therefore, an age-specific nomogram can improve the accuracy and practical value of the prediction model.

With the coming acceleration of global population aging, we are likely to witness a significant increase in the proportion of senile patients diagnosed with CHS, which will increase the public health burden. Consequently, based on publicly available data from the Surveillance, Epidemiology, and End Results (SEER) database, we aim to develop and validate a visual nomogram model to predict the survival probability of elderly patients with CHS. This study is expected to provide personalized survival predictions and optimize the clinical management of these patients.

## Materials and Methods

### Study Population

**Figure 1:**
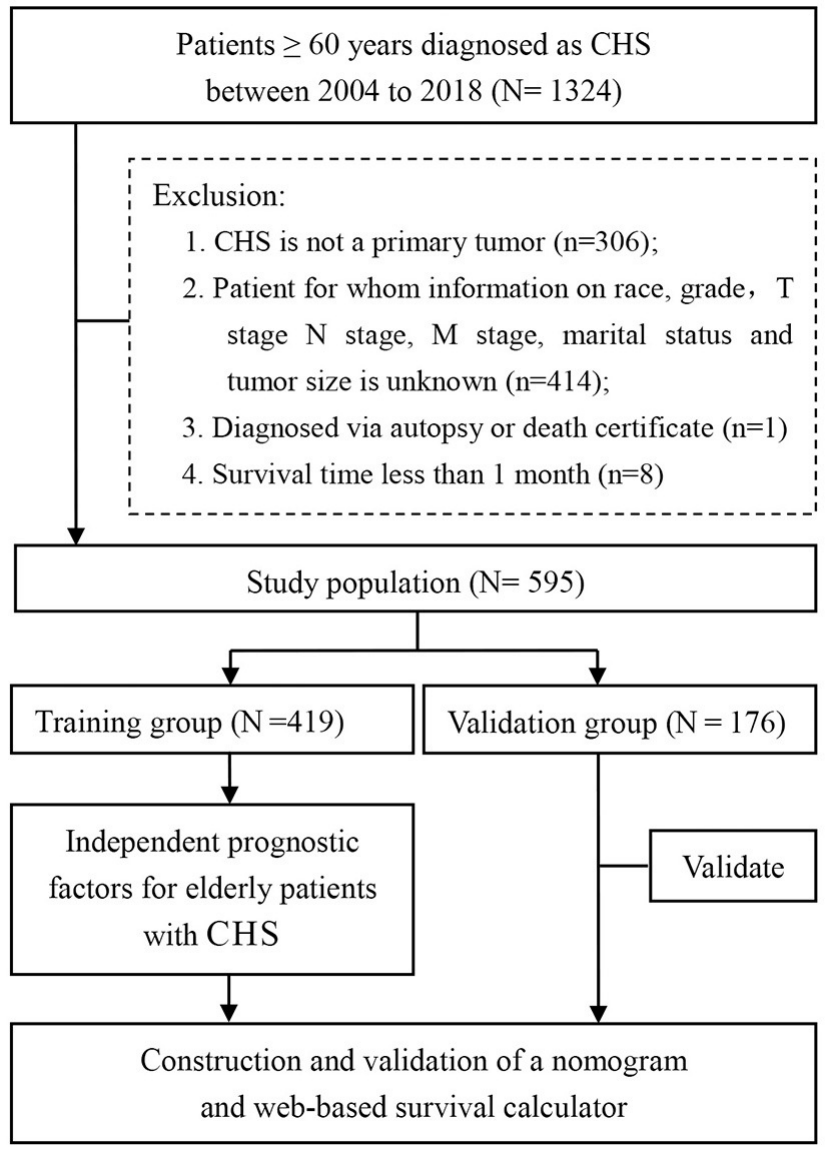
A total of 595 elderly patients with CHS were included in this study based on inclusion and exclusion criteria. After random assignment, the study was further analyzed.

The research data was obtained from the SEER-18 registries research database (www.seer.cancer.gov) using the SEER^*^Stat software (SEER^*^Stat 8.4.0) during the period of 2004 to 2018. Since the SEER database did not publish personally identifiable information, this study did not require the approval of the review committee and did not need to obtain informed consent. The inclusion criteria were outlined below: (1) patients were diagnosed with CHS (AYA site recode/WHO 2008: 4.2 Chondrosarcoma); (2) diagnosis years from 2004 to 2018; (3) patients with CHS aged ≥60 years at diagnosis; (4) completed follow-up. Also, there were four criteria for exclusion from the study, (1) CHS was not the first primary tumor; (2) patients whose variables included in analysis were unknown or blank; (3) patients who were diagnosed *via* autopsy or death certificate; (4) survival time <1 month. All enrolled cases were staged using the 8th edition of the AJCC TNM staging system. Figure 1 showed the workflow of this study.

### Data Selection

In this study, data extracted for each patient involved 14 variables. Demographic variables included age at diagnosis, sex, race, and survival time (months). And pathologic characteristics of the tumors included primary site, tumor size, and histology and AJCC TNM stage, in addition to their treatment information, including surgery, chemotherapy, and radiation, were obtained from the database. Among them, histology was divided into conventional, dedifferentiated, and myxoid. The age of the patients was divided into three groups, including <70 years, 70–80 years and >80 years. The optimal cutoff value for tumor size was evaluated using the X-tile software. Tumor size was classified on the basis of the largest tumor diameter (<55 mm, 55–150 mm and >150 mm). All screened eligible cases were composed of three types of primary site: axial bones, extremities and others. The primary endpoint of our study is overall survival (OS), which is defined as the time interval from the date of diagnosis to death from any cause.

### Statistical Analyses

All statistical analyses in this study were performed in SPSS 26.0 and R software (version 4.1.1). To ensure robustness and discrimination of the model, patients were randomly divided into a training group and a validation group at a ratio of 7:3 using the R software. Chi-square test and Fisher's exact test compared the baseline characteristics between the two groups. Variables associated with prognosis were determined by univariate Cox analysis. Then, variables with P<0.05 were included in the multivariate Cox analysis. Afterward, the independent prognostic factors of elderly patients with CHS were determined. The nomogram was developed based on the determined independent prognostic factors. The ability to discriminate between observed and predicted outcome was evaluated by Harrell's concordance index (C-index) (14). A higher C-index indicated a superior capacity to separate patients with different survival outcomes. Similarly, the receiver operating characteristic (ROC) curve with the value of area under curve (AUC) was further utilized to appraise the prediction efficiency of the model. And a *k*-fold cross-validation method was performed to validate the newly proposed model, with *k* = 10. In addition, the consistency between the predicted and actual survival was graphically assessed with the calibration curve performed according to a bootstrapped resample with 1,000 iterations. In addition, decision curve analysis (DCA), net reclassification improvement (NRI) and integrated discrimination improvement (IDI) (15) were used to assess the clinical usefulness of the nomogram and explore whether the model was more accurate than AJCC TNM staging system or not. Besides, the web-based survival calculator was further prepared based on the nomogram using the “Dynnom” package. Finally, the total point for all patients is calculated. Then, X-tile software is used to find the best cutoff value for the total point, patients in both groups were then classified into high, medium and low risk groups. Kaplan–Meier analysis with log-rank test was conducted to compare the survival differences between three subgroups. *P*-value < 0.05 (two-sided) was considered as statistically significant.

## Results

### Clinicopathologic Characteristics

According to the inclusion and exclusion criteria, 595 patients were finally included to this study. All patients were randomly divided into training group (419 cases) and validation group (176 cases). The training and validation groups had no significant difference (*p* > 0.05). Table 1 described the baseline data of the patients. Among the included cases, 53.61% of the patients were men, and 46.39% of the patients were women. The race distribution was predominantly white (92.61%). In terms of tumor characteristics, elderly patients with CHS were often Grade II (43.87%), N0 (98.82%), M0 (92.10%), and conventional (77.48%). Most patients underwent surgical resection (90.25%), while the minority received radiotherapy (16.30%) and chemotherapy (7.56%). Most patients were married (67.73%). More than half of the patients experienced a tumor size of 55–150 mm (56.97%).

**Table 1:** Clinicopathological characteristics of elderly patients with CHS.

**Variables**	**Total,** ***N* = 595**	**Training group,** ***N* = 419 (%)**	**Validation group,** ***N* = 176 (%)**	***p*-value**
**Age**
<70	327 (54.96)	229 (54.65)	98 (55.68)	0.5838
70–80	197 (33.11)	143 (34.13)	54 (30.68)	
>80	71 (11.93)	47 (11.22)	24 (13.64)	
**Race**
Black	20 (3.36)	13 (3.10)	7 (3.98)	0.7871
Other	24 (4.03)	16 (3.82)	8 (4.55)	
White	551 (92.61)	390 (93.08)	161 (91.48)	
**Sex**
Female	276 (46.39)	192 (45.82)	84 (47.73)	0.7377
Male	319 (53.61)	227 (54.18)	92 (52.27)	
**Marital status**
Married	403 (67.73)	289 (68.97)	114 (64.77)	0.3658
Unmarried	192 (32.27)	130 (31.03)	62 (35.23)	
**Primary site**
Extremity	294 (49.41)	198 (47.26)	96 (54.55)	0.1667
Axial	255 (42.86)	190 (45.35)	65 (36.93)	
Other	46 (7.73)	31 (7.40)	15 (8.52)	
**Grade**
Grade I	145 (24.37)	103 (24.58)	42 (23.86)	0.7183
Grade II	261 (43.87)	187 (44.63)	74 (42.05)	
Grade III	109 (18.32)	77 (18.38)	32 (18.18)	
Grade IV	80 (13.45)	52 (12.41)	28 (15.91)	
**Histology**
Conventional	461 (77.48)	331 (79.00)	130 (73.86)	0.2362
Dedifferentiated	98 (16.47)	62 (14.80)	36 (20.45)	
Myxoid	36 (6.05)	26 (6.21)	10 (5.68)	
**Tumor size**
<55 mm	185 (31.09)	133 (31.74)	52 (29.55)	0.8599
55–150 mm	339 (56.97)	237 (56.56)	102 (57.95)	
>150 mm	71 (11.93)	49 (11.69)	22 (12.50)	
**T stage**
T1	314 (52.77)	227 (54.18)	87 (49.43)	0.4606
T2	274 (46.05)	188 (44.87)	86 (48.86)	
T3	7 (1.18)	4 (0.95)	3 (1.70)	
**N stage**
N0	588 (98.82)	416 (99.28)	172 (97.73)	0.2337
N1	7 (1.18)	3 (0.72)	4 (2.27)	
**M stage**
M0	548 (92.10)	392 (93.56)	156 (88.64)	0.0623
M1	47 (7.90)	27 (6.44)	20 (11.36)	
**Surgery**
No	58 (9.75)	38 (9.07)	20 (11.36)	0.4779
Yes	537 (90.25)	381 (90.93)	156 (88.64)	
**Radiotherapy**
None	498 (83.70)	350 (83.53)	148 (84.09)	0.9627
Yes	97 (16.30)	69 (16.47)	28 (15.91)	
**Chemotherapy**
No	550 (92.44)	393 (93.79)	157 (89.20)	0.0779
Yes	45 (7.56)	26 (6.21)	19 (10.80)	

### Independent Prognostic Factors in Elderly Patients With CHS

**Figure 2:**
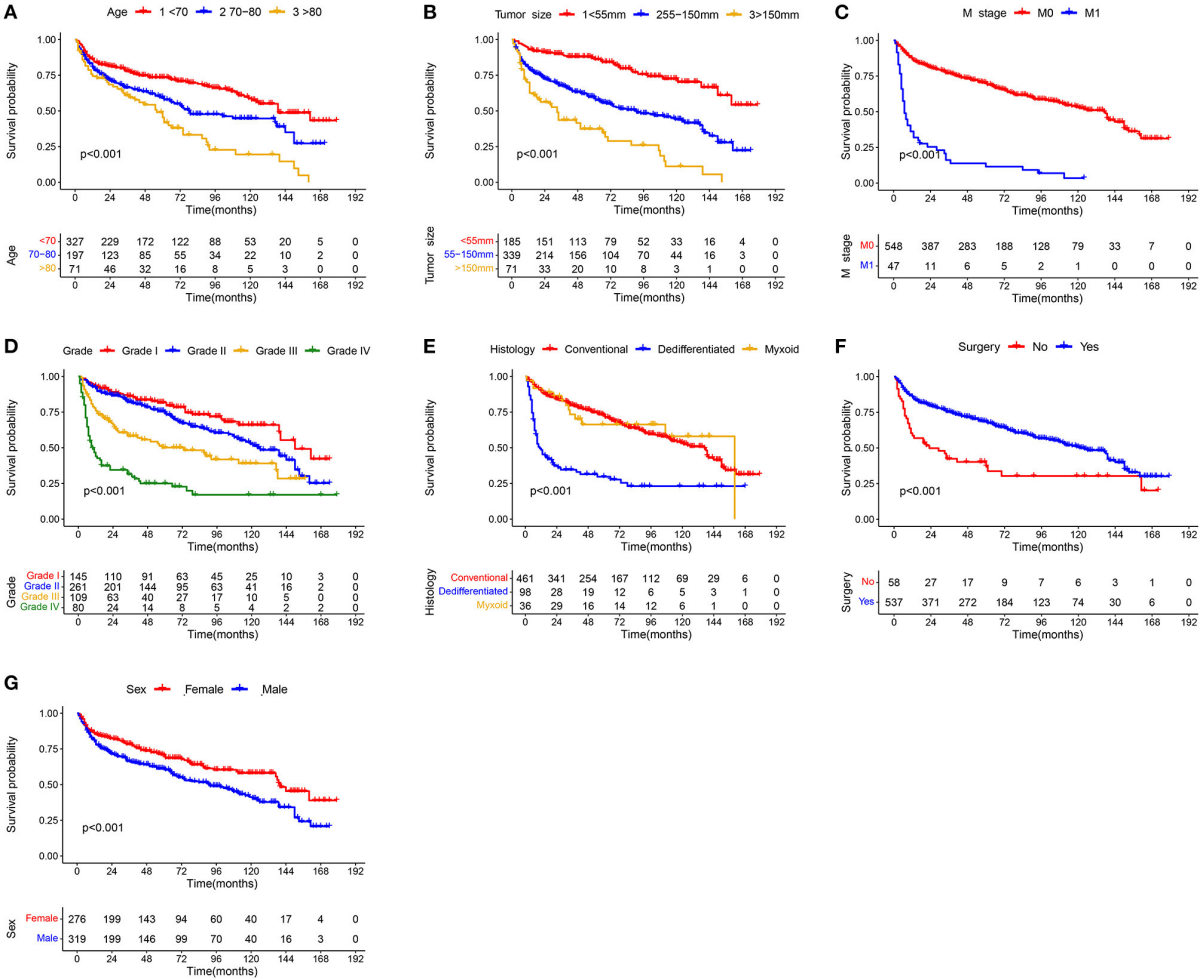
Kaplan–Meier survival curves of variables were performed for elderly patients with CHS: **(A)** age, **(B)** tumor size, **(C)** M stage, **(D)** grade, **(E)** histology, **(F)** surgery, and **(G)** sex.

In the univariate Cox analysis, 11 variables were found to be associated with OS of elderly patients with CHS, including age, sex, grade, histology, T stage, N stage, M Stage, surgery, radiotherapy, chemotherapy and tumor size (all *p*-value < 0.05). Then, multivariate Cox analysis was performed and seven variables were finally determined as independent prognostic factors, including age, sex, grade, histology, M stage, surgery and tumor size (Table 2). Consistent with univariate Cox analysis and multivariate Cox analysis, Kaplan–Meier survival analysis also showed that these variables (age, sex, grade, histology, tumor size, M stage and surgery) were significantly associated with OS (Figure 2).

**Table 2:** Univariate and multivariate Cox proportional hazards regression analysis of the OS of elderly patients with CHS.

**Variables**	**Univariate analysis**		**Multivariate analysis**	
	**HR (95% CI)**	***p*-value**	**HR (95% CI)**	***p*-value**
**Age**
<70 years	Reference		Reference	
70–80 years	1.79 (1.28–2.51)	0.001	1.51 (1.06–2.15)	0.0239
>80 years	3.15 (2.09–4.74)	<0.001	3.20 (2.07–4.95)	<0.001
**Sex**
Female	Reference		Reference	
Male	1.56 (1.15–2.13)	0.005	1.39 (1.01–1.91)	0.0459
**Race**
Black	Reference			
Other	0.91 (0.31–2.73)	0.873		
White	0.79 (0.35–1.79)	0.568		
**Grade**
Grade I	Reference		Reference	
Grade II	1.52 (0.97–2.38)	0.066	1.34 (0.84–2.15)	0.2181
Grade III	3.04 (1.87–4.94)	<0.001	1.67 (0.95–2.92)	0.0757
Grade IV	6.71 (4.07–11.06)	<0.001	3.49 (1.9–6.41)	<0.001
**Histology**
Conventional	Reference		Reference	
Dedifferentiated	4.56 (3.18–6.53)	<0.001	1.74 (1.1–2.77)	0.0189
Myxoid	0.97 (0.49–1.91)	0.926	0.68 (0.33–1.42)	0.3051
**Primary site**
Extremity	Reference			
Axial	0.79 (0.58–1.07)	0.129		
Other	0.61 (0.31–1.22)	0.164		
**T stage**
T1	Reference		Reference	
T2	2.76 (2.02–3.78)	<0.001	1.14 (0.74–1.75)	0.5662
T3	1.9 (0.46–7.79)	0.371	1.13 (0.26–4.9)	0.8666
**N stage**
N0				
N1	6.99 (2.21–22.07)	0.001	0.78 (0.22–2.78)	0.7042
**M stage**
M0	Reference		Reference	
M1	5.61 (3.62–8.72)	<0.001	2.77 (1.67–4.61)	<0.001
**Marital status**
Married	Reference			
Unmarried	1 (0.72–1.37)	0.979		
**Tumor size**
<55 mm	Reference		Reference	
55–150 mm	2.79 (1.84–4.24)	<0.001	1.76 (1.05–2.95)	0.033
>150 mm	6.37 (3.83–10.6)	<0.001	3.01 (1.51–6.03)	0.0018
**Surgery**
No	Reference		Reference	
Yes	0.45 (0.29–0.68)	<0.001	0.54 (0.32–0.9)	0.0187
**Chemotherapy**
No	Reference		Reference	
Yes	3.73 (2.37–5.86)	<0.001	1.51 (0.89–2.56)	0.1261
**Radiotherapy**
No	Reference		Reference	
Yes	1.64 (1.13–2.37)	0.009	1.35 (0.87–2.09)	0.1767

### Development and Validation of the Nomogram

**Figure 3:**
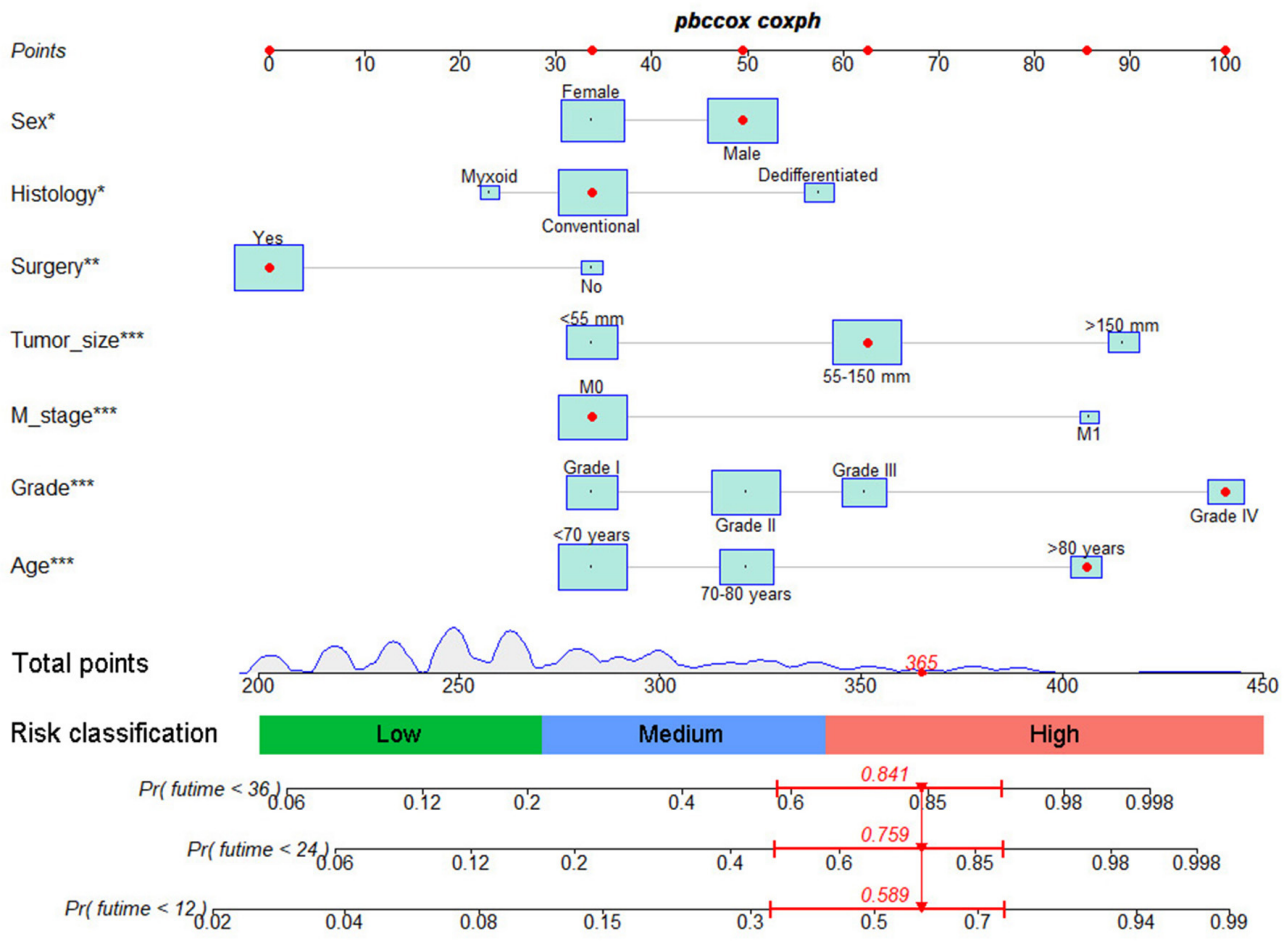
The graph showed nomogram for predicting 12-, 24-, and 36-month OS of elderly patients with CHS.

**Figure 4:**
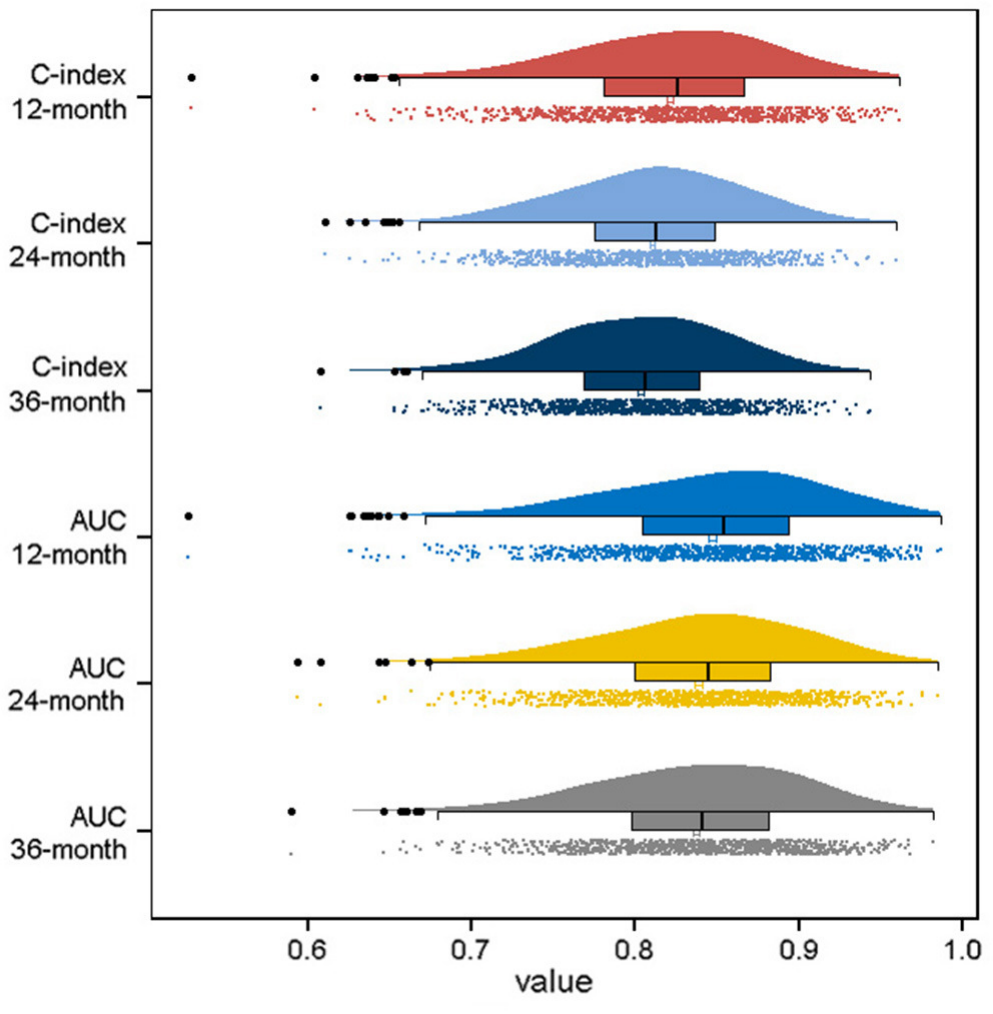
Visualization of the result of *k*-fold cross-validation (*k* = 10) through with half violin plot, scatter plot and boxplot with median.

**Figure 5:**
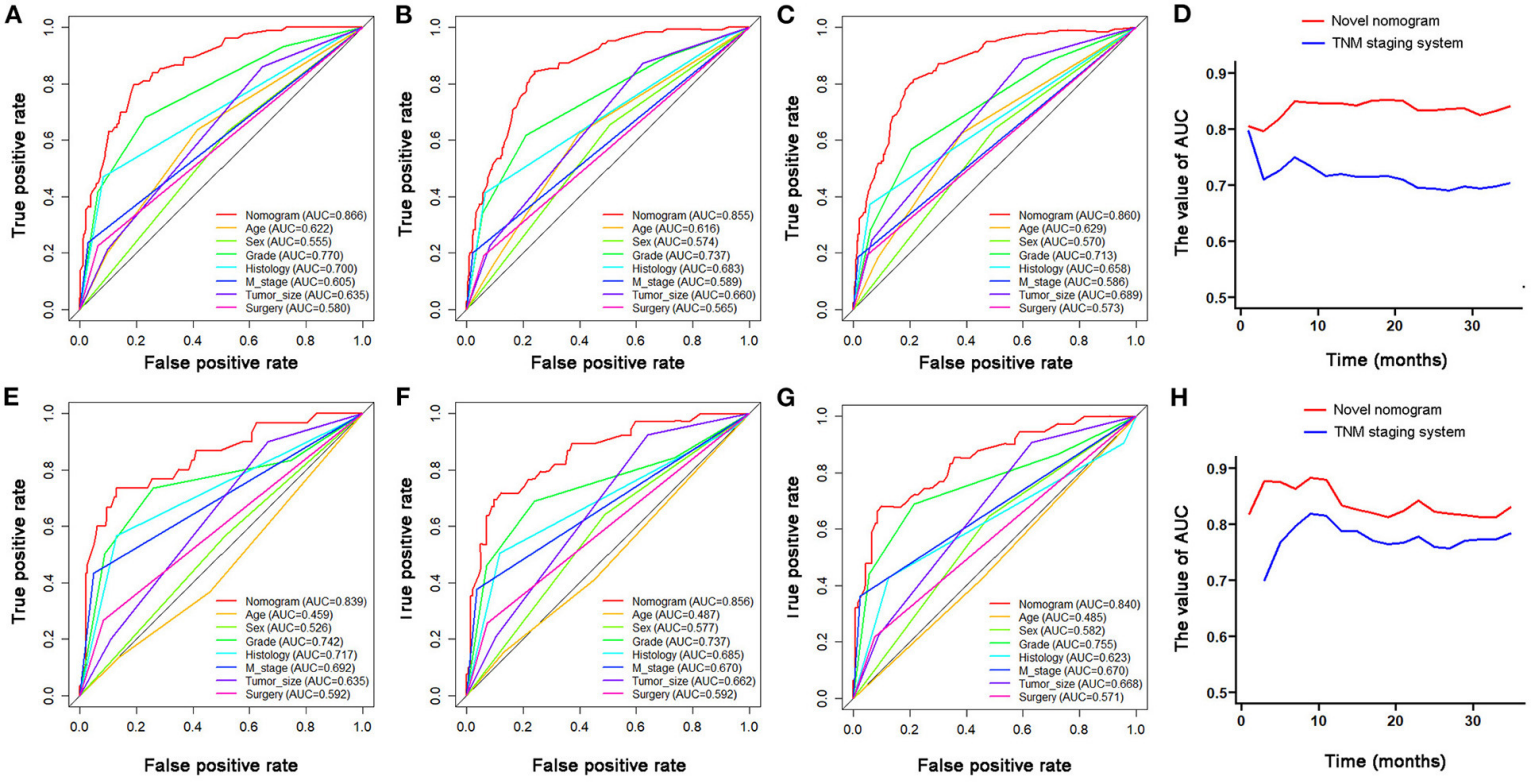
The comparison of ROC curves between nomogram and independent predictors at 12- **(A)**, 24- **(B)**, 36-month **(C)**, in the training group and at 12- **(E)**, 24- **(F)**, and 36-month **(G)** in the validation group. The time-dependent ROC curves of the nomogram for OS prediction in training group **(D)** and validation group **(H)**.

**Figure 6:**
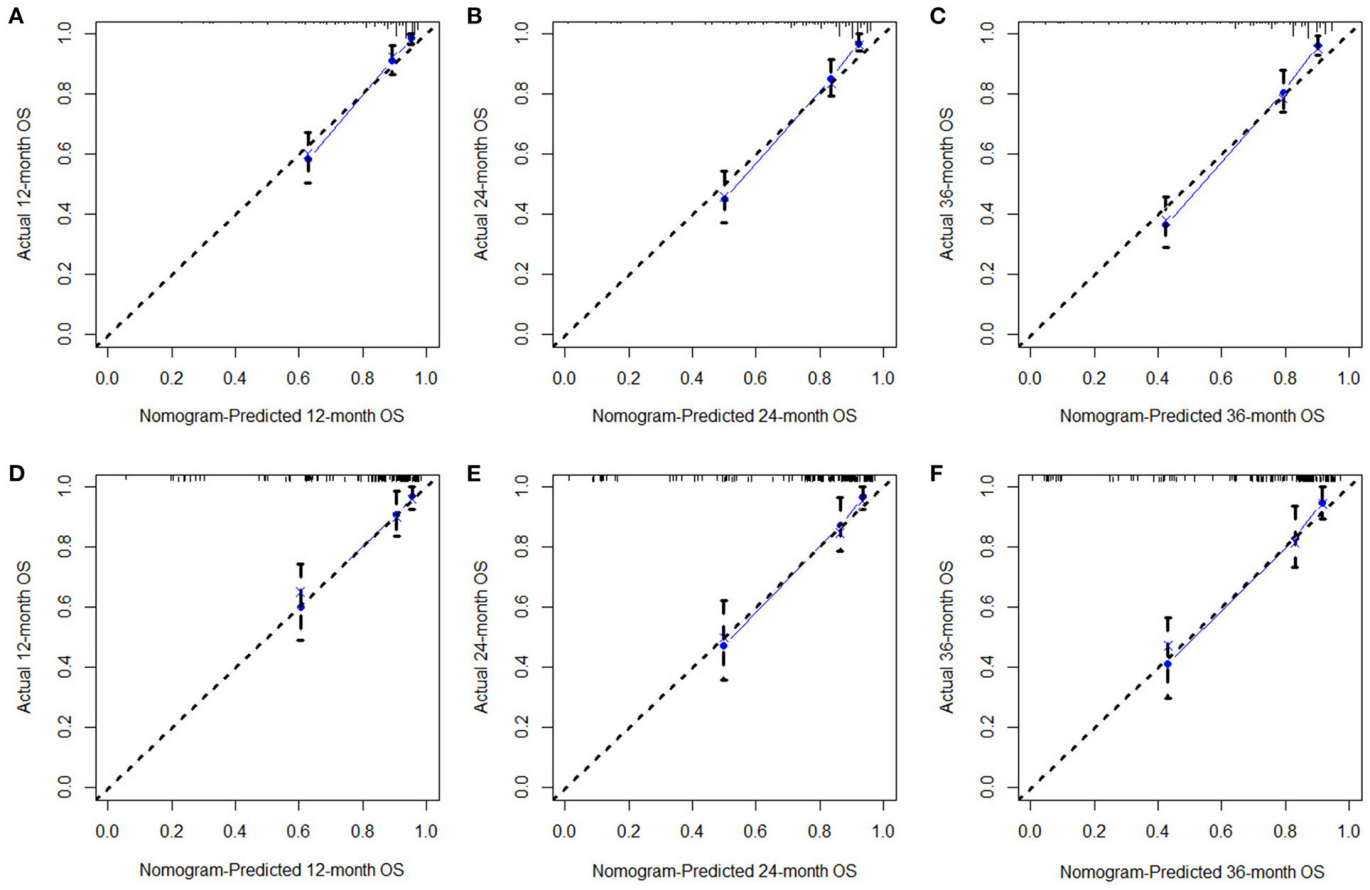
The calibration curves of 12- **(A)**, 24- **(B)**, 36-month **(C)**, OS in the training group and 12- **(D)**, 24- **(E)**, and 36-month **(F)** OS in the validation group. The dashed line represents an excellent match between nomogram prediction (X-axis) and actual survival outcome (Y-axis). The cohort was divided into five groups with equal sample size for internal validation. Closer distances from the points to the dashed line indicate higher prediction accuracy.

Based on the identified independent prognostic factors, we developed a nomogram to predict 12-, 24-, and 36-month OS of elderly patients with CHS (Figure 3). The overall performance of the nomogram was assessed, producing a C-index of 0.800 (95%CI: 0.733–0.867) in the training group and 0.789 (95%CI: 0.683–0.895) in the validation group, indicating the adequate discriminative ability of this prediction model. The result of *k*-fold cross validation (*k* = 10) indicated that the values of AUC for 12-, 24-, and 36-month were 0.847, 0.839, and 0.838 (Figure 4). Besides, the ROC curve showed that the value of AUC at 12-, 24-, and 36-month reached 0.866, 0.855, and 0.860 in the training group and of 0.839, 0.856, and 0.840 in the validation group, respectively, which meant a good distinguishing ability of this model. And the comparison of ROC curves between the nomogram and each prognostic factor indicated that the comprehensive model had higher discrimination than any single variable in both two groups (Figures 5A–C,E–G). In addition, the calibration curves for the training and validation groups showed a high degree of agreement between the actual observed results and those predicted by the nomogram (Figure 6).

### Comparison of the Nomogram and AJCC TNM Staging System

**Figure 7:**
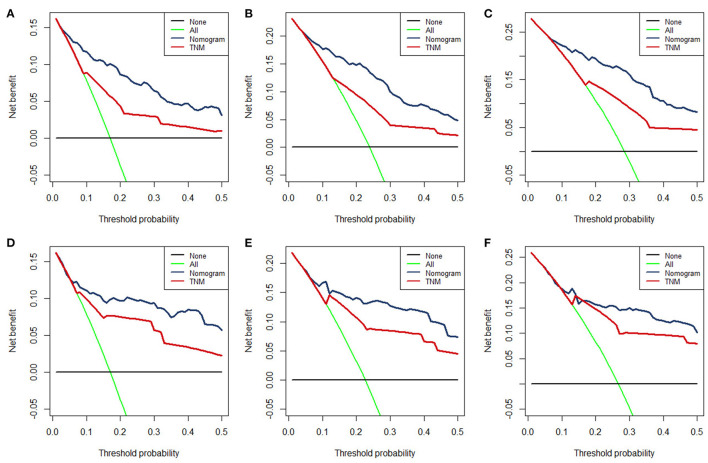
The DCA for 12- **(A)**, 24- **(B)**, 36-month **(C)**, OS prediction in training group and for 12- **(D)**, 24- **(E)**, and 36-month **(F)** OS prediction in the validation group.

The time-dependent ROC curve showed that the discrimination ability of the nomogram was better than the AJCC TNM staging system in both training and validation groups (Figures 5D,H). DCA analysis showed that compared with the traditional staging system, the net benefit of the newly proposed model was significantly increased and had a wide range of threshold probabilities (Figure 7).

In the accuracy analyses of NRI and IDI, the nomogram remained better performance than TNM staging system (Table 3). In the training group, the 12-, 24-, and 36-month NRI of the nomogram was 0.374, 0264, and 0.345, respectively. And the 12-, 24-, and 36-month IDI of the nomogram was 0.078, 0.102, and 0.115, respectively. In the validation group, the 12-, 24-, and 36-month NRI of the nomogram was 0.508, 0.236, and 0.235, respectively. And the 12-, 24-, and 36-month IDI of the nomogram was 0.070, 0.083, and 0.090, respectively. These results together demonstrated that the new nomogram had a superior predictive ability when compared with the conventional AJCC staging model (Table 3).

**Table 3:** NRI and IDI of the nomogram in survival prediction for elderly patients with CHS compared with TNM staging system.

**Index**	**Training group**			**Validation group**		
	**Estimate**	**95%CI**	***p*-value**	**Estimate**	**95%CI**	***p*-value**
**NRI (vs. AJCC TNM staging)**
For 12-month OS	0.374	0.181–0.548	<0.001	0.508	0.355–0.647	<0.001
For 24–month OS	0.264	0.175–0.476	<0.001	0.236	0.007–0.463	<0.001
For 36-month OS	0.345	0.167–0.544	<0.001	0.235	0.002–0.452	<0.001
**IDI (vs. AJCC TNM staging)**
For 12-month OS	0.078	0.053–0.103	<0.001	0.070	0.027–0.113	<0.001
For 24-month OS	0.102	0.073–0.131	<0.001	0.083	0.036–0.130	<0.001
For 36-month OS	0.115	0.084–0.146	<0.001	0.090	0.043–0.137	<0.001

### Ability of Nomogram to Stratify Patient's Mortality Risk

**Figure 8:**
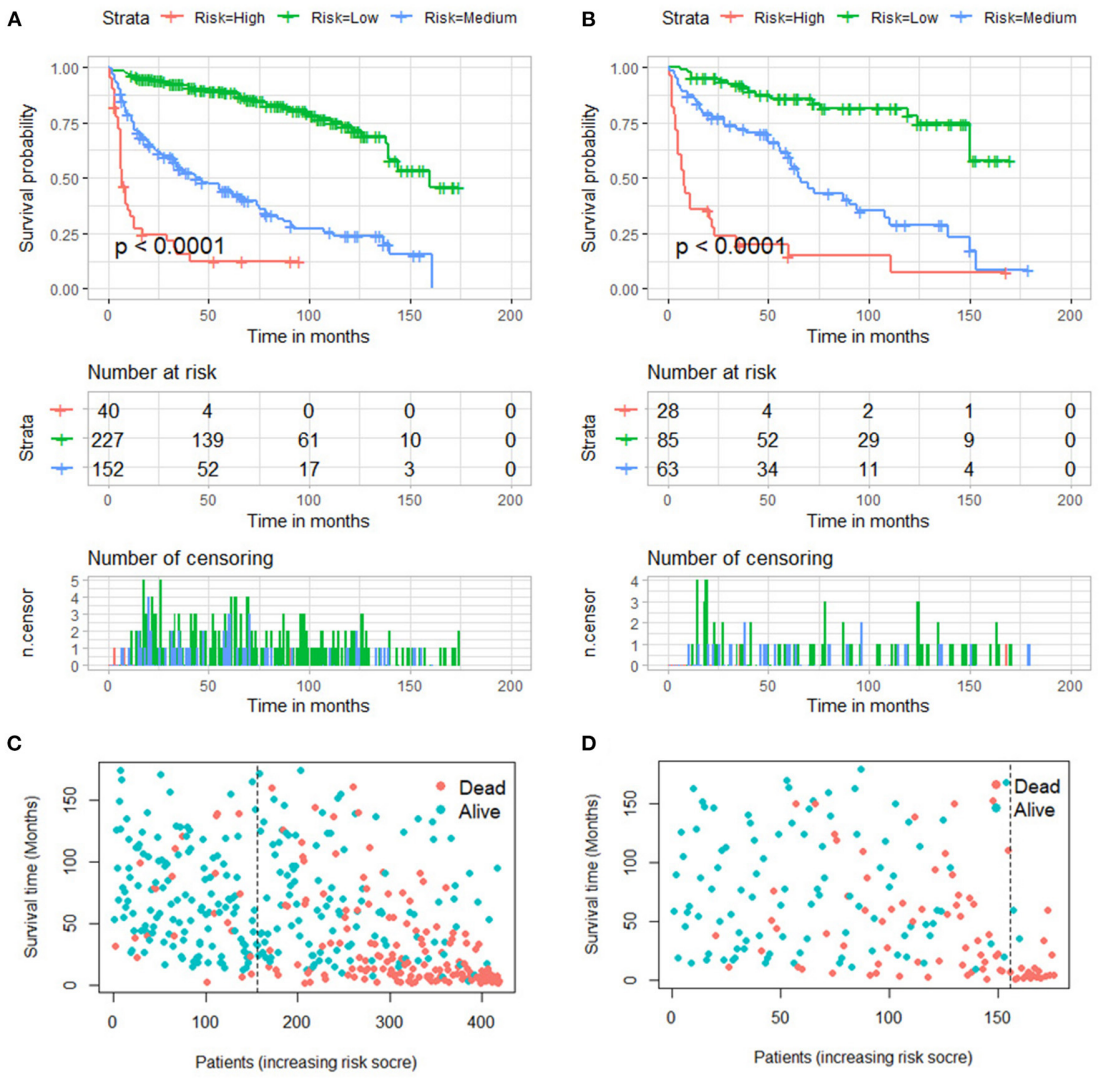
Kaplan–Meier survival curves of three mortality risk subgroups in the training group **(A)** and validation group **(B)**. Kaplan–Meier survival status analysis in the training group **(C)** and validation group **(D)**.

Furthermore, total point for all patients was calculated according to the nomogram. The best cutoff values were determined as 270 and 341 by X-tile software. Subsequently, the patients in two groups were both divided into low (total point <270), medium (270 ≤ total point ≤ 341) and high (total point > 341) mortality risk subgroups, a. As shown in Figure 8, the Kaplan–Meier survival analysis with log-rank test suggested that there was a statistically difference (*P* < 0.001) in comparing the survival curves for all three subgroups in both training and validation groups. Patients with high-risk scores had a worse prognosis than those with low-risk scores.

### Development of a Dynamic Web-Based Calculator

**Figure 9:**
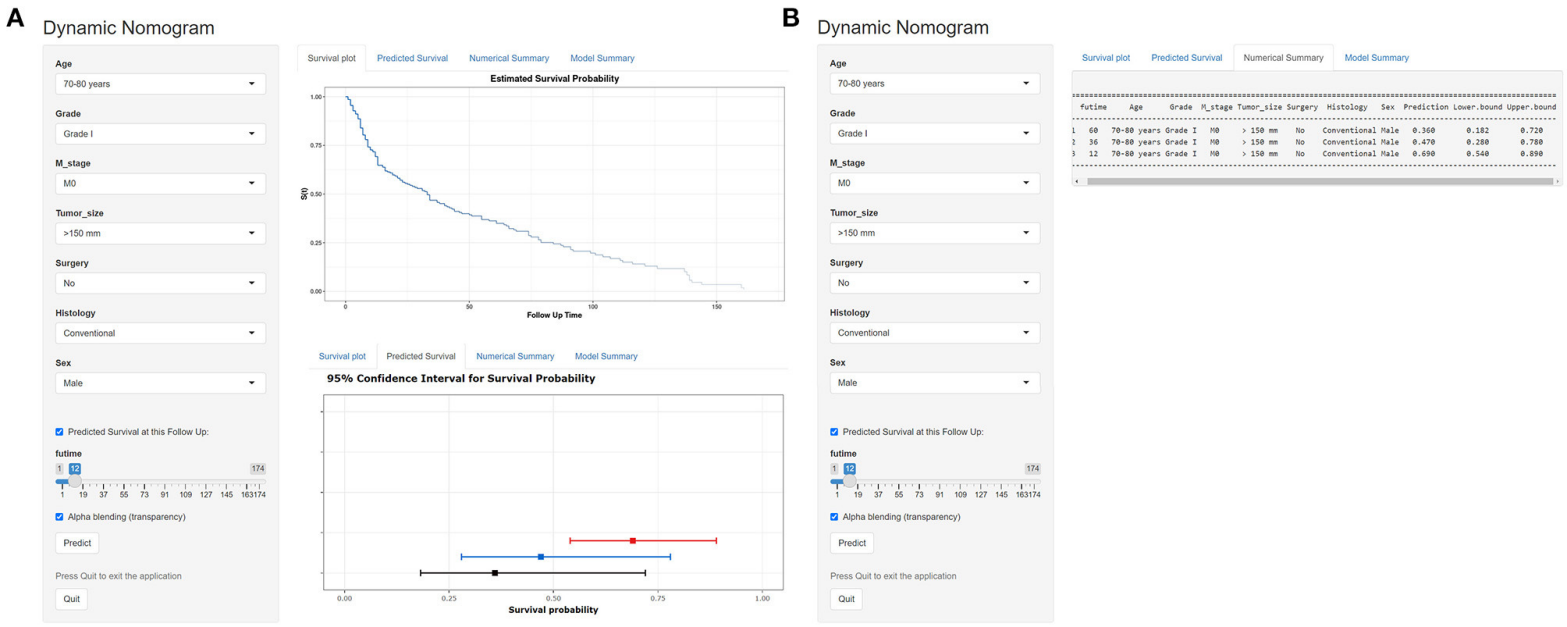
Operation interface of nomogram on web page. After entering a patient's age, sex, M stage, tumor size, surgery, histology, and grade on the web, a clinician can ascertain a patient' s OS, survival probability. **(A)** The graphical summary of predicted OS and 95% CI at 12 months (red), 24 months (blue), and 36 months (black). **(B)** The specific numbers of survival rate.

According to the established nomogram, we further developed a dynamic web-based survival calculator to simplify application of this nomogram (available from https://nomoresearch.shinyapps.io/elderlywithCHS/) (Figure 9). With the web-based survival rate calculator that we built, we were better able to evaluate our patients in the clinic, and thus contribute to better treatment. It is convenient to predict survival probability and its 95% CI by inputting their clinical features. For instance, for a 72-year-old male patient with M0, grade I, tumor size of 155 mm, histology of conventional, without surgery, the 5-year OS rate was ~36.0% (95% CI, 18.2–72.0%).

## Discussion

It has been reported that ~3,610 patients with primary malignant bone tumors were newly diagnosed in the United States in 2021 (16). Of these, the number of patients with CHS is the second highest and becomes more common with age (3, 17). Age is generally considered to be closely related to tumor prognosis. Nie et al. reported that the survival rate of patients with CHS worsened with age (18). Another study suggested that the poor prognosis of elderly patients with CHS may be related to differences in tumor characteristics (19). With the change of demographic structure, the burden of cancer among the elderly has become even heavier (20). This undoubtedly poses a new challenge to the clinical management of elderly patients with CHS. Following these considerations, elderly patients with CHS deserve to be treated as a representative population for individualized exploration of prediction of survival probability.

The nomogram is a user-friendly visual statistical model that incorporates multiple predictors and provides individualized survival prediction for clinicians and patients. It is extensively applied to a wide range of malignancies owing to its ease of use and reliable discriminative power (21–23). The nomogram for predicting survival in patients with CHS was established for the first time in the study of Song et al. (11). In a recent study, Zheng et al. investigated the prognostic factors and the difference between different surgery scopes in sacrum/pelvic chondrosarcoma patients, subsequently, a nomogram was constructed (24). Nevertheless, their study neither highlighted the elderly population nor compared the newly constructed nomogram with the traditional AJCC TNM staging system, thus, there is still uncertainty about the accuracy of survival prediction. Furthermore, it is also inconvenient to use only graphical predictive model in a clinical setting. In a retrospective study by Liu et al., they specially focused on elderly patients with osteosarcoma and developed a prognostic nomogram for this population (25). However, to the best of our knowledge, there is still lack of analyses of prognostic factors and survival trends elderly patients with CHS. To better address this issue, we used a population-based database to identify independent prognostic factors and develop a web-based nomogram to predict survival rate of elderly patients with CHS.

In this study, we identified seven independent predictors associated with prognosis in elderly patients with CHS, including age, sex, grade, histology, M stage, surgery and tumor size. The result indicated that CHS patients in the age group of >80 years faced the worst OS (HR: 3.20, 95% CI: 2.07–4.95), which again confirmed that advanced age was positively associated with a worse prognosis in CHS patients. Worse nutritional status, further reduction in physiological reserve, more complex underlying disease and poor tolerance to treatment might be explanations for the poorer survival rate of CHS patients as they aged. On the other hand, older patients are prone to immune senescence, which allows tumors to evade surveillance by the immune system, consequently, primary tumor in elderly patients with CHS tend to be more aggressive at the time of initial diagnosis (26, 27). Besides, we found that sex was also significant prognostic factors, among elderly patients diagnosed with CHS, men experienced worse survival outcomes than women, which was which is similar to a previous report (28).

The characteristics of the tumor tend to affect its prognosis. Low-grade CHS is generally regarded as an indolent tumor, while high-grade CHS is usually detrimental to patient survival (5). Boehme et al. reported that patients with high-grade CHS had poorer prognosis, with a 5-year survival rate of only 50–60% (29). Poor differentiation is considered to be more aggressive, a feature that leads to a higher risk of local recurrence and metastasis, which definitely has a negative impact on the survival outcome of patients. The histology of CHS is also closely related to its prognosis. Amer et al. studied the survival and prognosis of five known non-conventional subtypes of CHS based on the SEER database, they found that the dedifferentiated subtype was responsible for the lowest median survival of only 11 months, while the while the juxtacortical subtype had the highest, with 97 months (7). Similarly, Song et al. suggested that histology had a significant impact on the prognosis of CHS, the survival rate of patients with conventional CHS was better than that of patients with dedifferentiated CHS (11). Consistent with the previous reports, we found that histology was an independent prognostic factor in elderly patients with CHS. A few of published studies showed that tumor stage and tumor size could predict the prognosis of patients (30, 31). In this study, our results also indicated that M stage and tumor size were independent prognostic factors for elderly CHS patients. The presence of distant metastases at the time of initial diagnosis meant that the disease had progressed to an advanced stage and cancer treatment was less effective (32). At the same time, larger tumors might represent a longer period of tumor growth, which increased the possibility of metastasis and made complete surgical resection difficult.

The efficacy of chemotherapy and radiotherapy for CHS remained controversial in view of its low percentage of dividing cells and poor vascular distribution (33, 34). Surgical resection was the gold standard for the treatment of primary or recurrent CHS (35, 36). In the present study, we found that the prognosis of patients treated with surgery was significantly different from those treated with radiotherapy or chemotherapy. After univariate and multivariate analyses, only surgical treatment was finally determined as an independent prognostic factor for elderly patients with CHS, while radiotherapy and chemotherapy were excluded. We may need to note, however, that complete surgical resection was specific to older patients diagnosed as CHS, due to the fact that more than half of cancer patients over 65 years of age suffered from the double burden of the cancer itself and other coexisting chronic diseases (37).

Based on the independent prognostic factors discussed above, we also constructed a prediction model with excellent performance. It can quantify the probability of OS for elderly patients with CHS by combining the determined independent predictors. Furthermore, after evaluating the predictive accuracy and clinical usefulness of the model, the results of ROC curves, DCA, NRI, and IDI together demonstrated that the newly proposed nomogram had a superior predictive ability when compared with the existing AJCC staging model. Another strength of this study was that a web-based survival calculator had also been developed to facilitate the clinical application of the model. A patient' s survival probability with 95% CI can be easily obtained after the values of seven variables and time have been input in the webpage of https://nomoresearch.shinyapps.io/elderlywithCHS/.

It was undeniable that this study still had some limitations. First, as a retrospective study, potential selection bias was inevitable. Second, if another independent large-scale data was used for external verification, the result may be more reliable. Third, the nomogram provided a relative reference for clinicians. Other factors related to the prognosis of elderly patients with CHS might exist in the clinical setting.

## Conclusion

This study found that age, sex, grade, histology, M stage, surgery, and tumor size were independent prognostic factors for elderly patients with CHS. The web-based nomogram model can accurately predict OS of elderly patients with CHS. It was expected to inform clinical decision making and help develop targeted treatment strategies for this population.

## Data Availability Statement

The raw data supporting the conclusions of this article will be made available by the authors, without undue reservation.

## Ethics Statement

We received permission to access the research data file in the SEER program from the National Cancer Institute, US. Approval was waived by the Ethics Committee of China-Japan Union Hospital of Jilin University, as SEER data is publicly available and de-identified. All methods were carried out in accordance with relevant guidelines and regulations.

## Author Contributions

YT and DZ conceived and designed the study and revised the manuscript. YT and YC collected the clinical data and literature review. YT and LJ conducted the statistical analysis. YT, YP, and YG generated the figures and tables. YT wrote the manuscript. DZ supervised the research. All authors critically read the manuscript to improve intellectual content. All authors read and approved the final manuscript.

## Conflict of Interest

The authors declare that the research was conducted in the absence of any commercial or financial relationships that could be construed as a potential conflict of interest.

## Publisher's Note

All claims expressed in this article are solely those of the authors and do not necessarily represent those of their affiliated organizations, or those of the publisher, the editors and the reviewers. Any product that may be evaluated in this article, or claim that may be made by its manufacturer, is not guaranteed or endorsed by the publisher.

## Abbreviations

CHS: chondrosarcoma
OS: overall survival
SEER: Surveillance Epidemiology and End Results
AJCC: American Joint Committee on Cancer
CI: confidence interval
HR: hazard ratio
ROC: receiver operate curve
AUC: area under curve
DCA: decision curve analysis
C-index: Harrell's concordance index
NRI: net reclassification improvement
IDI: integrated discrimination improvement.
